# Rolling the DICE (Design, Interpret, Compute, Estimate): Interactive Learning of Biostatistics With Simulations

**DOI:** 10.2196/52679

**Published:** 2024-04-15

**Authors:** Robert Thiesmeier, Nicola Orsini

**Affiliations:** 1 Department of Global Public Health Karolinska Institutet Solna Sweden

**Keywords:** learning statistics, Monte Carlo simulation, simulation-based learning, survival analysis, Weibull

## Abstract

Despite the increasing relevance of statistics in health sciences, teaching styles in higher education are remarkably similar across disciplines: lectures covering the theory and methods, followed by application and computer exercises in given data sets. This often leads to challenges for students in comprehending fundamental statistical concepts essential for medical research. To address these challenges, we propose an engaging learning approach—DICE (design, interpret, compute, estimate)—aimed at enhancing the learning experience of statistics in public health and epidemiology. In introducing DICE, we guide readers through a practical example. Students will work in small groups to plan, generate, analyze, interpret, and communicate their own scientific investigation with simulations. With a focus on fundamental statistical concepts such as sampling variability, error probabilities, and the construction of statistical models, DICE offers a promising approach to learning how to combine substantive medical knowledge and statistical concepts. The materials in this paper, including the computer code, can be readily used as a hands-on tool for both teachers and students.

## Introduction

The correct use and application of statistics plays a fundamental role in the health sciences, in turn providing objective and quantitative evidence to support decision-making in public health [1]. Despite the increasing relevance of statistics in health research, it is often taught in isolation, usually through standard lectures covering the theory and methods followed by computer exercises with given data sets. This can lead to a disconnect between statistical and epidemiological methods such as study design, as well as insufficient awareness of important statistical concepts such as sampling variability [2]. Therefore, teaching methods that deliver statistical concepts in conjunction with epidemiology for students in the health sciences are crucial for educational development [3].

Simulation-based learning has previously been proposed as a tool to support engaging learning [4] and has been shown to be an effective learning method to develop critical thinking and reflective skills [5-7]. In the context of public health and epidemiology, 2 articles in particular highlight Monte Carlo simulations [8] (hereafter simulations) as a method to illustrate, learn, and understand statistical and epidemiological concepts. First, Rudolph et al [3] demonstrate how to use simulations to teach and learn nondifferential misclassification and understand the concept of the *P* value. Second, Fox et al [9] illustrate how to design simple simulations from directed acyclic graphs and use them to explain epidemiological concepts. Both papers provide helpful resources for students to familiarize themselves with the basics of setting up a simulation.

However, despite a broad acceptance of simulations as a helpful tool to learn statistical and epidemiological concepts [5], in our experience, they are rarely implemented as the main teaching and learning method for students in health sciences. Rather than using simulations to learn a stand-alone element of statistics, we propose a learning method that uses simulations to explore and understand the major steps involved in conducting a scientific investigation. In expanding upon the current foundations of simulation-based learning in health sciences, we introduce DICE (design, interpret, compute, estimate), an engaging, problem- and simulation-based learning method. The overall aim is to promote statistical reasoning in the health sciences by combining medical and statistical knowledge in designing epidemiological studies. The purpose of this viewpoint paper is therefore to describe the concept of DICE and discuss its potential strengths and limitations in learning statistics in the health sciences. The statements expressed in this paper are based on the experiences and opinions of the authors.

The remaining part is structured as follows: we will first describe the proposed method—DICE—and explain the intended learning objectives and outcomes. We will then illustrate the use of DICE with an example of a time-to-event outcome. Finally, we will discuss some potential strengths and limitations of applying the method in a classroom setting.

### The DICE Approach

DICE is an engaging learning method that enables students to use simple simulations to design, analyze, and interpret a realistic epidemiological study (note that the acronym DICE represents the learning steps involved, but not in order). The use of DICE as a learning tool combines problem-oriented learning [10,11] with simulations [12]. A detailed description of Monte Carlo methods can be found elsewhere [13]. While there are numerous ways to simulate artificial data, we focus on the approach presented by Fox et al [9] due to its simplicity to implement in statistical software and its easy-to-follow translation from a causal framework. In brief, simulations enable us to study a mechanism empirically by sampling from a statistical model that governs the mechanism. Data are then sampled from a predefined probability distribution (eg, Bernoulli, normal, or Weibull) that defines the mechanism, commonly referred to as the inverse transformation sampling method [14]. Further, the data are analyzed with an appropriate statistical model (preferably the same model that generated the data). These steps can be repeated a large number of times to empirically observe the variability of the sampling process [13].

### Steps and Learning Objectives

**Figure 1:**
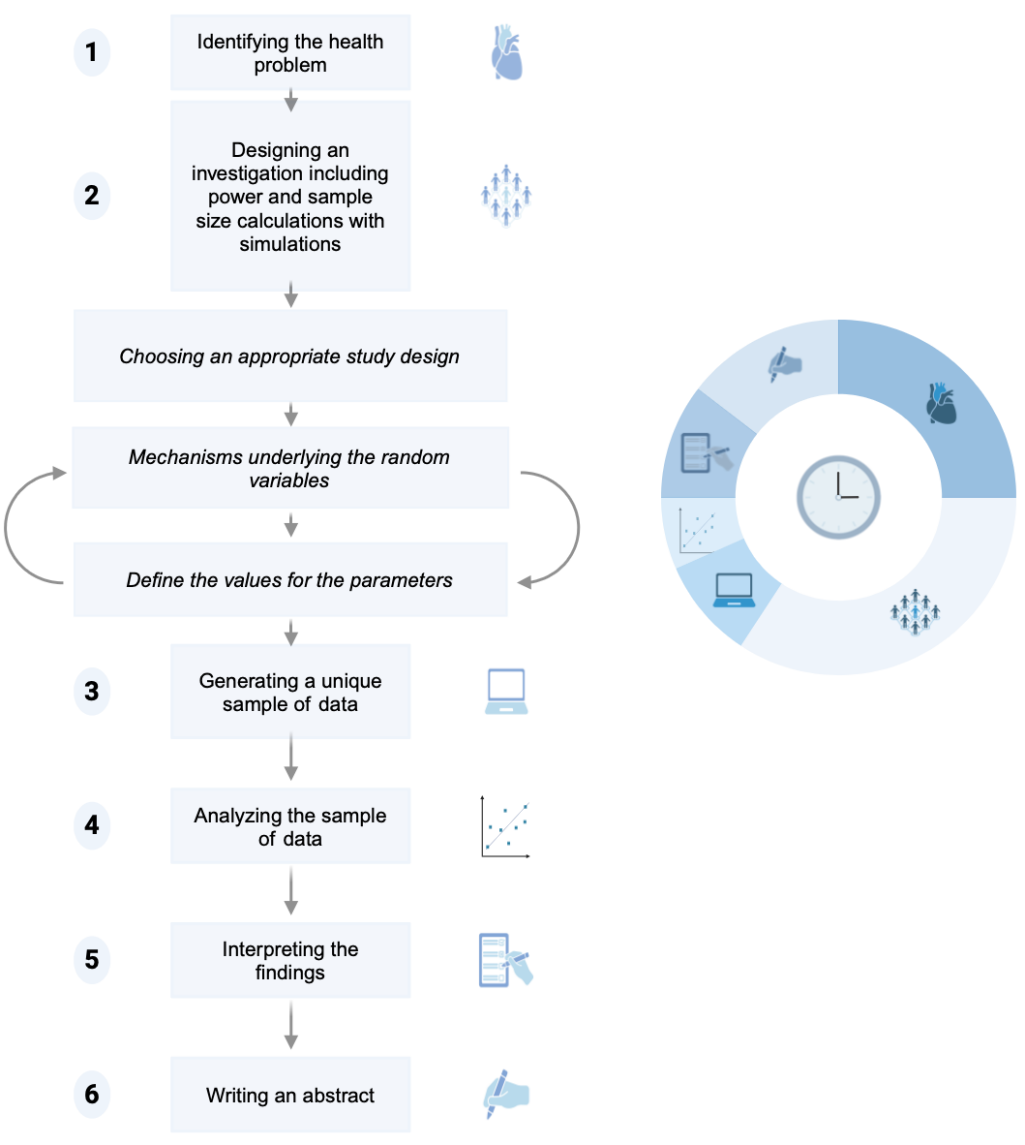
Flowchart of the 6 steps involved in DICE (design, interpret, compute, estimate) with an illustrative pie chart showing the approximate time dedicated to each step. The curved arrows in step 2 indicate that these activities can be completed multiple times to calibrate the sample size and power of a study before moving on to the next step.

DICE includes 6 major steps that cover the major stages of a scientific investigation. Figure 1 visualizes the chronological order of these steps and highlights the approximate amount of time that one step requires. The second step—designing an investigation including power and sample size calculations with simulations—is further divided into 3 parts, which can be repeated to calibrate the power and sample size of a study before moving on to step 3.

**Figure 2:**
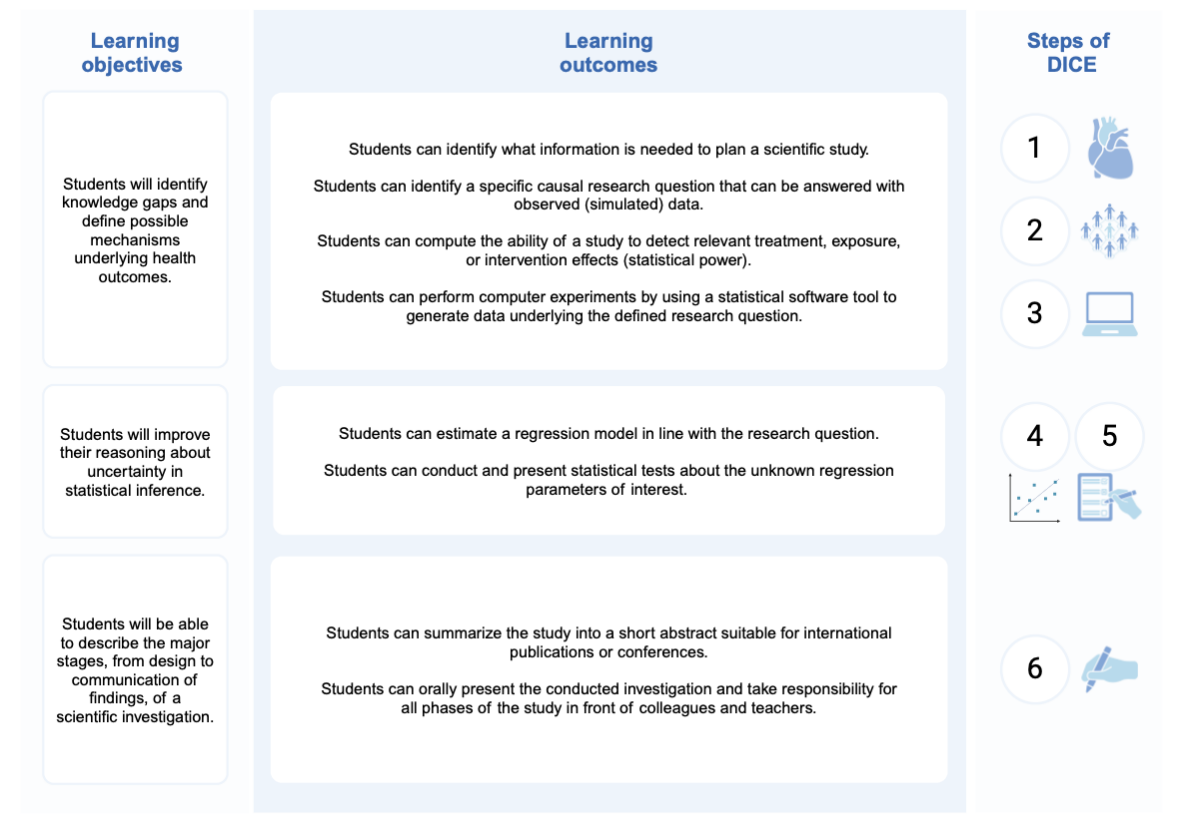
The main learning objectives and outcomes across the steps of DICE (design, interpret, compute, estimate). The steps of DICE include (1) identifying the health problem, (2) designing an investigation including power and sample size calculations with simulations, (3) generating a unique sample of data, (4) analyzing the sample according to the plan, (5) interpreting the findings carefully, and (6) writing a short abstract to be presented in class.

The key learning objectives and outcomes of DICE, as highlighted in Figure 2, target experiential learning [15] and active learning styles [16] according to Bloom’s taxonomy of educational objectives, including applying recently learned concepts and theories, making informed judgements and evaluations, and generating new knowledge [17]. DICE is a flexible method that accommodates different learning styles that have been shown to play an important role in medical education [18]. As such, each student can work according to their strengths (eg, taking a leading role in the group to cover a specific aspect of the design of a simulated study, like computer coding or result interpretation). Due to the heterogeneity in the working groups, it can be expected that students will use their own learning styles and strengths to learn from other students with different skills [18].

### A Guide Through an Example

Each step is now practically explained with an example. The following example is inspired by 2 recent epidemiological studies [19,20]. All information and data are simulated and only serve educational purposes. The computer code in Stata (StataCorp) and R (R Core Team) can be readily used to replicate the example (the code is provided in Multimedia Appendix 1).

#### Step 1: Identifying the Health Problem

During the first step of DICE, students should think about a particular problem, population, and area that they would like to investigate. This can be somewhat time-consuming and requires a decision about the nature of the research question (ie, causal, descriptive, or predictive) [21]. As we focus on simulating data according to a causal framework explained by Fox et al [9], the research questions are intended to answer a causal question. Other forms of research questions can, of course, be incorporated and simulated; however, they are not the focus of this example. In our example, the aim is to examine the effect of physical activity on the 10-year mortality rate in a large cohort of older people.

#### Step 2: Designing an Investigation Including Power and Sample Size Calculations With Simulations

The second step addresses the overall design of the study, including the assessment methods for the specified variables. The step is further divided into three specific parts: (1) students should reflect on the appropriate study design (eg, experimental or observational), (2) put forward the possible mechanisms (confounding, interaction, etc) underlying all the random variables involved in the study, and (3) discuss plausible values for all of the parameters. These are discussed in more detail below.

##### Part 1: Choosing an Appropriate Study Design

Designing an investigation that includes power and sample size calculations with simulations requires careful consideration of available literature and substantive knowledge about the underlying health problem. We recommend allocating sufficient time for this step of planning a realistic simulation study.

In our example, we design a large, observational cohort study with a confounding effect by age. Information on physical activity (3.5 hours per week of moderate to vigorous physical activity [MVPA] vs less), together with age (≥80 years vs <80 years), is assessed at baseline in a short questionnaire. The mortality rate in a cohort of older people is likely to increase over time due to aging, among both physically active and inactive populations. Assuming a baseline mortality rate in the younger and physically inactive population of 7 deaths per 1000 person-years, we determined that 5000 individuals (about 1005 deaths during 10-year follow-up) would provide a statistical power of about 86% to detect at least a 20% lower mortality rate (age-adjusted hazard ratio 0.8) in the physically active population relative to the inactive population. A 2-sided Wald-type test for the age-adjusted hazard ratio conferred by physical activity equal to 1 with a type II error of 5% is conducted based on a multivariable Weibull survival model including physical activity and age as covariates.

**Figure 3:**
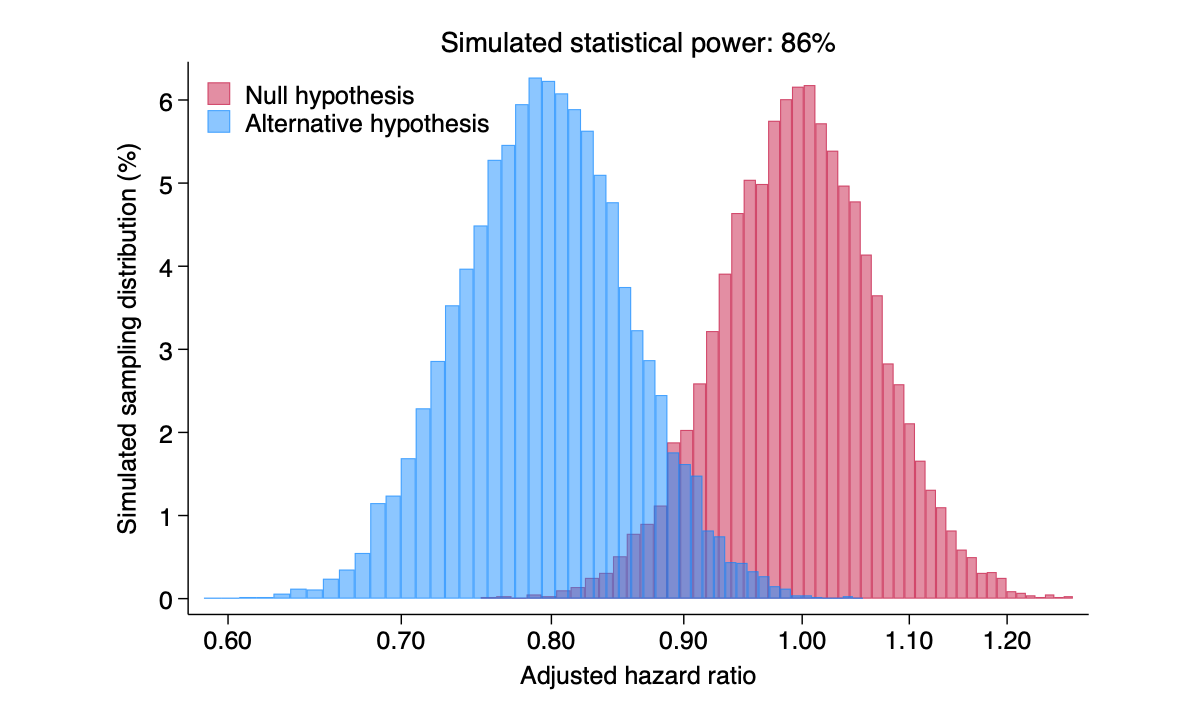
Simulated sample distribution of the age-adjusted mortality hazard ratio comparing active versus inactive individuals under the null and alternative hypotheses (hazard ratio 0.8). The simulated statistical power was obtained by counting the number of studies that correctly rejected the null hypothesis with a 2-sided Wald-type test at a significance level of 5% based on a multivariable Weibull survival model. The number of simulations is 10,000, the sample size of each study is 5000, and the average number of deaths within each study is 1005.

Figure 3 shows the sampling distribution of the age-adjusted hazard ratio comparing physically active versus inactive individuals under the null and alternative hypotheses.

##### Part 2: Mechanisms Underlying the Random Variables

Parameters and their distributions can be inspired by previous studies, textbooks, or substantive knowledge from group members. For example, if the exposure is defined as systolic blood pressure (mmHg), students can assume an approximately symmetric and bell-shaped distribution with a given mean and SD and derive the parameter from a normal distribution function. For this study, we need the following variables: (1) *z*, an indicator variable for the older population (1 “>80” vs 0 “≤80 years”); (2) *x*, an indicator variable for the physically active population (1 “>3.5 h/w of MVPA” vs 0 “≤3.5 h/w MVPA”); and (3) *t*, the time from baseline to death (in years) or the end of follow-up (10 years), whichever came first.

##### Part 3: Define the Values for the Parameters

During this step, students should write a few lines of code or a function capable of generating data according to the desired study and mechanism. Simulations can be used to calibrate the sample size and statistical power of the study. To achieve the desired statistical power (eg, 80%), the sample size can be changed accordingly during this step. This requires some time, and we recommend students try to adapt certain values for the parameters or underlying mechanisms from the previous step (Figure 1**)**. This process is commonly referred to as the data generating mechanism (DGM). We understand DGM as the mechanism underlying the causal structure, including the uncertainty governing the observed data. The simulated power of the statistical test to detect an effect is simply given by the sum of studies that reject the null hypothesis of no effect divided by the total number of simulated studies.

In our example, the first variable to be generated is baseline age (about 60% are older than 80 years of age), which is a confounding variable in the relationship between physical activity and mortality:



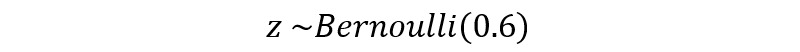



For the exposure model, the second variable to be generated is baseline physical activity as a function of age. People aged ≤80 years have a probability of being physically active of 50%, whereas the odds of being physically active among older people are 1/3 (67% lower odds) relative to younger people:



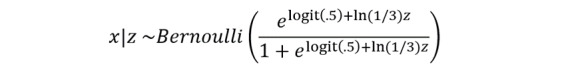



Of note, Bernoulli is a statistical function, whereas logit and ln are a mathematical function.

For the outcome model, individual time-to-death (in years) conditional on the variables physical activity and age is obtained under the Weibull survival model, as follows:



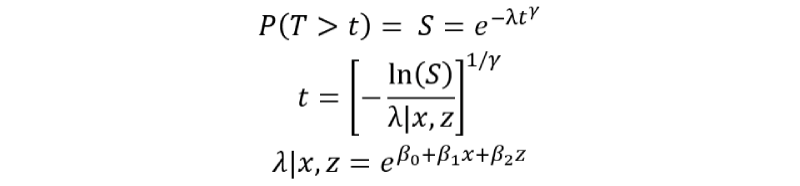



where γ is the parameter defining the departure from a simpler exponential (constant mortality rate) survival model. The value of γ is set to 1.1, indicating a slight increase in the mortality rate over the follow-up period in all the covariate patterns.

The natural logarithm of the baseline mortality rate (per 1 year) among young and inactive people is assumed to be 7 deaths per 1000 person-years, so the intercept is *β*_0_=ln(7/1000)=–4.962

The age-adjusted mortality hazard ratio comparing physically active versus inactive people is set to 0.80, as determined by *β*_1_=ln(0.80)=–0.2231.

The physical activity-adjusted mortality hazard ratio comparing older versus younger people is set to 4, computed as *β*_2_=ln(4)=1.386.

Given a random value for the survival probability *S* ranging over the 0 to 1 interval, a random value of individual time-to-death (in years) conditionally on the variables physical activity and age is obtained as follows:



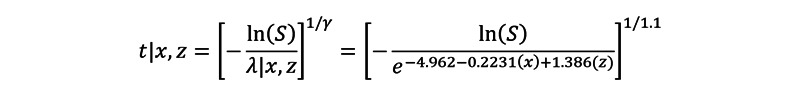



In addition, any randomly generated time-to-death beyond the follow-up time of 10 years is set to 10 and considered censored (ie, still alive at the end of the follow-up). An indicator variable for death or censored status is also created to inform any survival analysis.

#### Step 3: Generating a Unique Sample of Data

Once the study has been designed with sufficient statistical power to detect the relevant effect, the next step is to draw one unique sample. Students will analyze and present only this sample in class. The uniqueness and reproducibility of the simulated data are guaranteed by setting a numerical sequence, called a seed, before obtaining realizations of the random variables. This is important for the exact replication of the study. Every group of students is asked to use a common seed in generating the analytical sample of data so that all groups replicate the study under the same conditions. Each group will have a different research question and an underlying health problem with varying parameters. The reason for choosing a seed in the beginning is to highlight the uniqueness of a single study generated under a known DGM. The easiest choice is to specify the seed according to the date of the DICE activity. In our example, we use the seed 20230413 (based on the year, month, and date: “YYYYMMDD”). However, for specific tasks such as power calculations or simulating a distribution of effects, the seed must be deleted to ensure variability in the simulations.

#### Step 4: Analyzing the Sample of Data

The outcome model is specified according to the process underlying the data, and it is estimated based on the only sample available. Students estimate the statistical model whose performance was evaluated in the initial step of the study design. In our example, we estimate a multivariable Weibull regression model including physical activity and age as covariates.

#### Step 5: Interpreting the Findings

Students carefully interpret the estimated model and write about the inferential results. In our example, during the 10-year follow-up period, a total of 974 people died out of 5000. Compared with inactive people, the age-adjusted hazard ratio for active people was 11% lower (hazard ratio=0.89; 95% CI 0.78-1.03). A Wald-type 2-sided test indicates some compatibility between this sample of data and the hypothesis of a null age-adjusted mortality hazard ratio for physical activity (*z*=–1.52; *P*=.13). This unique sample of data is an example of type II error (failing to reject the null hypothesis, which is indeed incorrect). Nonetheless, the magnitude and direction of the hazard ratio indicate a beneficial effect of physical activity on the 10-year mortality rate. This provides an example of correctly differentiating statistical and scientific inference.

#### Step 6: Writing an Abstract

Each group of students should then write a structured scientific abstract (200-250 words) summarizing all the previous steps suitable for an epidemiological conference. The findings and interpretation are then presented in class. Each group of students briefly presents their findings and reasoning behind the study design. Teachers and peers have the possibility of asking questions. The presentations of each group should not exceed 10 minutes per group.

### What Have we Learned?

**Figure 4:**
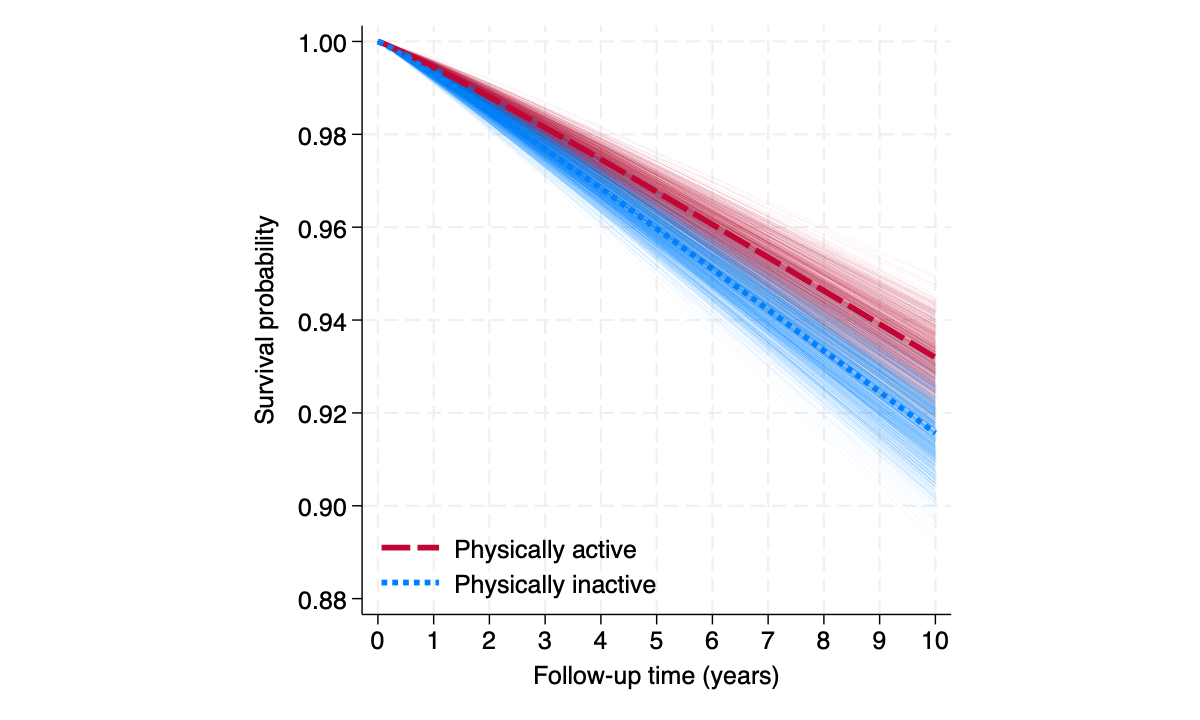
Sampling variability of the estimated survival probability comparing physically active and physically inactive young participants based on 900 simulated studies. The thick dashed lines for both physically active and inactive groups show the functions that were set under the original data-generating mechanism.

Based on our experiences teaching with DICE and to conclude the steps of DICE shown in the practical example, we hope the key learning lessons for students will include the following: First, students should realize that the most challenging and time-consuming step is the design of the study and identifying a plausible distribution of the random variables involved, the mechanisms underlying the data, and all the parameters included. Second, students should understand that error probabilities (type I, type II, and power) in conducting a test of hypothesis can be easily evaluated by replicating the study many times under similar conditions (Figure 3) using a simulation. Third, students should appreciate the fundamental distinction between the analysis of a single study and the analysis of a collection of estimates obtained from its replication (Figure 4). Fourth, students should understand that the ability of a study to find a relevant exposure or intervention effect (statistical power) can be achieved only with respect to one parameter of interest. Fifth, students should learn that the correct use of statistics plays a key role in all stages of a scientific investigation.

### Strengths and Limitations of DICE

We proposed an engaging learning method, DICE, to stimulate experimental, active, and enjoyable learning of statistical concepts, fostering key scientific skills in designing and conducting experiments. While the main strengths of this approach lie in its interactivity and group-based nature, we acknowledge several limitations.

First, the proposed simulation method is practically limited to only a few numbers of parameters that can be included in the design of a study. Each additional variable increases the complexity of the DGM exponentially. Thus, this approach is best suited for illustrative, simplified examples of realistic health problems. More sophisticated data derived from multivariate distributions would exceed the simplicity of the method but can, of course, be considered for more advanced classes.

Second, implementing DICE is resource-intensive and should not be done in a short time frame (eg, less than 1 hour). Although this is not a direct limitation of the method, it might be a limitation of its implementation in a classroom.

Third, the effectiveness of DICE in conveying statistical concepts in epidemiology has not been formally evaluated yet. This paper is a description and discussion of the method as implemented in class at a medical university. A formal evaluation of its effectiveness in learning statistics is being devised.

### Implementing DICE in the Classroom

Based on the experiences of the authors in using DICE, we summarize the following points for its implementation in the classroom for graduate students in medical sciences, including public health and epidemiology.

First, to implement DICE in a classroom, we recommend a classroom size of approximately 20-40 students, with small groups of 3-5 students from heterogeneous scientific backgrounds. Each group should consist of students who have different strengths and learning styles. We experienced that this could improve interaction between students and increase the joy of learning statistics.

Second, throughout the group work, students are encouraged to discuss and reflect upon the study design, practice the generation and simulation of data under a certain mechanism, and communicate their findings and interpretation of the study. We experienced that some students require more support to understand and use the provided computer code, particularly in settings with fewer students experienced in coding. It can help to go through an example of a simulated study with Stata or R code in front of the class.

Third, DICE can be implemented within a full day of teaching or over several days. For a 1-day implementation, the morning can be used for students to frame their research question and develop the study using simulations (steps 1-3). The afternoon can then be reserved for steps 4-6, ending with the presentation of the abstracts. It is important to keep in mind that the first 2 steps require most of the time (Figure 1). Students should not be rushed through these steps and should be provided with sufficient guidance and support to find an adequate research question, study design, and set up the simulations. Alternatively, DICE can be implemented over several days. An introduction to DICE is given in class, and students can work over several days in their respective groups. The final day can be used for presenting and discussing the studies and outcomes of each group.

### Conclusion

This paper introduces an engaging simulation-based method, DICE, to learn statistics in the health sciences. We argue that DICE can boost statistical reasoning and bridge the gap between substantive knowledge and statistics for all major steps of a scientific investigation. Students can learn fundamental statistical and epidemiological concepts with simulations and combine learning of technical aspects such as coding with theoretical concepts such as error probabilities. The materials in this paper can be readily used by teachers and students.

## Appendix

Multimedia Appendix 1 Computer code in Stata and R to replicate the example used in the paper.

## Abbreviations

DGM: data generating mechanism
DICE: design, interpret, compute, estimate

## Data Availability

All data generated or analyzed during this study are included in this published article and its supplementary information files.
