# Supplementary material for: Rolling the DICE (Design, Interpret, Compute, Estimate): Interactive Learning of Biostatistics With Simulations
Source: JMIR Med Educ. 2024 Apr 15;10:e52679. doi: 10.2196/52679 (PMC11058551; doi:10.2196/52679)
Supplement: Multimedia Appendix 1 [file mededu_v10i1e52679_app1.docx]

**Multimedia Appendix 1. Computer code in Stata and R to replicate the example used in the paper.**

* Generate a single study

clear all

set obs 5000

set seed 20230413

gen z = rbinomial(1, .6)

gen x = rbinomial(1, invlogit(logit(.5)+ln(1/3)*z))

scalar follow_up = 10

scalar b0 = ln(7/1000)

scalar b1 = ln(.80)

scalar b2 = ln(4)

scalar gamma = 1.1

gen time = (-ln(runiform())/exp(b0+b1*x+b2*z))^(1/gamma)

gen death = (time < follow_up)

replace time = follow_up if time > follow_up

stset time, fail(death)

streg x z, dist(weib)

* Generate many samples

capture program drop sim_weibull

program define sim_weibull, rclass

syntax[, obs(real 5000)]

drop _all

set obs `obs'

gen z = rbinomial(1, .6)

gen x = rbinomial(1, invlogit(logit(.5)+ln(1/3)*z))

scalar follow_up = 10

scalar b0 = ln(7/1000)

scalar b1 = ln(.80)

scalar b2 = ln(4)

scalar gamma = 1.1

gen time = (-ln(runiform())/exp(b0+b1*x+b2*z))^(1/gamma)

gen death = (time < follow_up)

replace time = follow_up if time > follow_up

stset time, fail(death)

streg x z, dist(weib)

ret scalar est_b1 = _b[x]

test x

ret scalar p_value = r(p)

end

simulate est_b1 = r(est_b1) p_value = r(p_value), ///

reps(10000) seed(20230413): sim_weibull

**R Code**

rm(list = ls())

library(eha)

logit <- function(p) {log(p/(1 - p))}

invlogit <- function(p){1/(1+exp(-p))}

# Generate a single study

n_obs <- 5000

z <- rbinom(n_obs, 1, 0.6)

x <- rbinom(n_obs, 1, invlogit(logit(0.5) + log(1/3) * z))

follow_up <- 10

b0 <- log(7/1000)

b1 <- log(0.8)

b2 <- log(4)

gamma <- 1.1

time <- (-log(runif(n_obs,0,1))/exp(b0 + b1 * x + b2 * z))^(1/gamma)

death <- (time < follow_up)

time[time > follow_up] <- follow_up

surv_obj <- Surv(time, death)

df <- data.frame(time, death, x, z)

model1 <- weibreg(Surv(time, death) ~ x + z, data = df)

summary(model1)

# Generate many samples

sim_surv <- function(){

n_obs <- 5000

z <- rbinom(n_obs, 1, 0.6)

x <- rbinom(n_obs, 1, invlogit(logit(0.5) + log(1/3) * z))

follow_up <- 10

b0 <- log(7/1000)

b1 <- log(0.8)

b2 <- log(4)

gamma <- 1.1

time <- (-log(runif(n_obs,0,1))/exp(b0 + b1 * x + b2 * z))^(1/gamma)

death <- (time < follow_up)

time[time > follow_up] <- follow_up

surv_obj <- Surv(time, death)

df <- data.frame(time, death, x, z)

model1 <- weibreg(Surv(time, death) ~ x + z, data = df)

est_b1 = coef(model1)[1]

est_se_b1 = sqrt(model1$var[1,1])

p_value = 2*pnorm(est_b1/est_se_b1)

cbind(est_b1, est_se_b1)

b = cbind(est_b1, p_value)

return(b)

}

sim_surv()

S <- 1000

est_b1 <- rep(NA, S)

p_value <- rep(NA, S)

for (i in seq_len(S)) {

est <- sim_surv()

est_b1[i] <- est[1]

p_value[i] <- est[2]

}
